# Editorial: Epigenetic regulation in cancer: mechanisms, implications, and therapeutic interventions

**DOI:** 10.3389/fcell.2026.1953900

**Published:** 2026-08-25

**Authors:** Angelo Sparaneo, Anthony K. N. Chan, Federico Pio Fabrizio

**Affiliations:** 1 Laboratory of Oncology, Fondazione IRCCS Casa Sollievo della Sofferenza, San Giovanni Rotondo, Foggia, Italy; 2 Department of Biochemistry and Molecular Biology, Mayo Clinic, Rochester, MN, United States; 3 Department of Medicine and Surgery, University of Enna “Kore”, Enna, Italy

**Keywords:** cancer epigenetics, DNA methylation, epitranscriptomics, non-coding RNAs, precision oncology

The field of rapidly evolution of cancer epigenetics is constantly shifting our knowledge about tumor biology, uncovering how modifiable changes that affect DNA, RNA, chromatin, and non-coding RNA (ncRNA) molecules affect the expression of different genes, which then drive cancer development, spread, and drug resistance (Sherif et al., 2025; Sadida et al., 2024). Beyond genetic mutations, epigenetic and epitranscriptomic modifications have emerge as essential factors involved in oncogenesis, providing exciting opportunities for the identification of biomarkers and novel therapeutic treatment strategies (Dai et al., 2024).

In July 2025, we successfully launched a highly relevant Research Topic titled “Epigenetic Regulation in Cancer: Mechanisms, Implications, and Therapeutic Interventions” in Frontiers in Cell and Developmental Biology, in which we integrated and combined various biological and molecular aspects taken from reviews, mini-reviews, original research articles, and short reports, which, taken together, highlighted the diversity and complexity of the epigenetic mechanisms involved in cancer and related diseases. The contributions range from DNA methylation to RNA modifications, ncRNA biology, regulation of the tumor microenvironment (TME), and emerging *in vitro* and preclinical strategies.

One aspect that certainly proved interesting in this Research Topic was the central role of ncRNAs in tumor development and progression. Thus, Lin and colleagues examined the involvement of microRNAs (miRNAs) in macrophage polarization and colorectal cancer, highlighting how miRNA-mediated regulation of tumor-associated macrophages may influence inflammatory responses and reshape the tumor microenvironment. To round out this perspective, Gan et al. have pointed the attention on the reciprocal interactions between hypoxia-inducible factors (HIFs) and long non-coding RNAs (lncRNAs) in gastrointestinal cancers. Their work demonstrates how hypoxia-driven lncRNA networks are involved in proliferation, metastasis, apoptosis, and drug resistance, emphasizing the importance of the adaptation of the microenvironment during cancer growth.

Of particular interest is the complexity of ncRNA regulation, as well described in the intriguing review by Ortega and collaborators, which examined the developmental and tumorigenic functions of the H19 imprinted maternally expressed transcript (H19)/Insulin-like Growth Factor 2 (IGF2) locus, that is subject to genetic imprinting. Their analysis showed that the loss of epigenetic imprinting mechanisms led to the reactivation of developmental pathways driving epithelial-mesenchymal transition, abnormal proliferation, and the spread of metastasis. Similarly, Gao and coworkers in a brief research report article provided new insights into the biological functions of parent gene *Membrane Palmitoylated Protein 6 (MPP6)* and its circular (circ)MPP6 in non-small cell lung cancer (NSCLC), underlining the notion that circRNAs should be considered as independent regulatory factors rather than solely as by-products of host gene expression.

Many of the articles in this Research Topic collection are focused on the fast-growing field of epitranscriptomics. In a review article, Ramalingam et al. have comprehensively summarized the interaction of N6-methyladenosine (m6A) modifications and noncoding RNAs, emphasizing how the interplay between these pathways may lead to tumor cell growth, immune modulation, and therapy resistance. Broadened this Research Topic, in a mini review, Wang and colleagues addressed the emerging roles of circRNA modified with m6A in lung cancer, exploring their involvement in tumor pluripotency, ferroptosis, metabolic reprogramming, and their influence on treatment sensitivity.

Further studies have expanded our understanding of RNA-modifying enzymes as key modulators of tumor biology. In another mini review, Li et al. summarized the physiological and pathological functions of Methyltransferase Like 5 (METTL5), an 18S rRNA methyltransferase which, when dysregulated, drives oncogenic protein translation, metabolomic adaptation, and reprogramming of the immune system. In addition to this, Zhou and collaborators explored NOP2/Sun RNA Methyltransferase 5 (NSUN5)-mediated m5C RNA methylation, pointing out its role in tumor progression, metabolomic reprogramming, the adaption to stress response, and evasion to immune system mechanisms. Likewise, Similarly, Xiong and coworkers investigated the multiple functions of NAT10 (N-acetyltransferase 10)-mediated N4-acetylcytidine (ac4C) modifications, focusing on their contribution to tumor growth, metastasis, and drug resistance, as well as their diagnostic and therapeutic potential. Collectively, these findings have highlighted how RNA modifications have been recognized as a fundamental layer of gene regulation in the development of cancer.

The intersection between epigenetic regulatory mechanisms and the immune landscape is also a central theme discussed in this research section. In a review paper, Duan and colleagues have critically evaluated advancements in RNA methylation within the context of gastrointestinal cancers and outlined how RNA-modifying enzymes may be involved in immune cell function, metabolic reprogramming, and immune evasion. Their paper highlights the possibility of combining epitranscriptomic and immunotherapy approaches in order to boost the effectiveness of treatment.

Moreover, Stoccoro et al. have delved into emerging aspects of epigenetic regulation, presenting an updated perspective on mitochondrial epigenetic mechanisms in cancers. Their review examined the methylation of mitochondrial DNA, non-coding mitochondrial RNAs, and cross-talk between nuclear and mitochondrial epigenetic networks, opening up new avenues for metabolic regulation and therapeutic vulnerabilities. At the same time, in a interesting review article, Baspakova and collaborators have tackled a highly cutting-edge and environmentally significant Research Topic by checking out the potential role of miRNAs in tackling the cancer-causing pathways associated with exposure to microplastics. While largely speculative, their *in silico* analysis may suggest new paths for future discovery-driven research.

This Research Topic also encompasses original studies that contribute to progress at the mechanistic and translational levels. In an exploratory study, Fabrizio and colleagues studied the methylation status of *SPARC* (Secreted Protein Acidic and Rich in Cysteine, a multifaceted matrix protein linked to extracellular matrix reorganization, cell-matrix interplay, tissue repair, and cell fate determination) in idiopathic pulmonary fibrosis (IPF), with a significant methylation increase in the promoter region with respect to non-fibrotic lung tissue. It is worth noting that the authors found an inverse correlation among promoter hypermethylation and SPARC expression, which was proven by demethylation assays that recovered gene expression in human primary fibrotic cells. These findings led to the conclusion that epigenetic silencing of *SPARC* may be responsible for the pathogenesis of the disease and might serve as a promising molecular biomarker for IPF.

Based on original research articles published with a focus on promising therapeutic targets and strategies, Liu et al. demonstrated that paeonol was able to suppress the progression of oral squamous cell carcinoma by repressing the phosphoinositide 3-kinase/protein kinase B (PI3K/AKT) signaling pathway as well as inducing protective autophagy, thereby illustrating that a combined approach targeting these pathways has the potential to increase anti-tumor efficacy. The findings reported in another original article by Shen and collaborators are also very interesting, in which they found that miR-495 represses osteosarcoma growth and metastasis by directly regulating Runt-related transcription factor 3 (RUNX3) and inhibiting PI3K/AKT signaling, underpinning the therapeutic potential of miRNA-based therapeutics.

Another original article that worthy of attention in the context of precision oncology approaches is that by Dzikowski and colleagues, who have shown that pharmacological inhibition of Protein Arginine Methyltransferase 5 (PRMT5) drives a functional Ataxia Telangiectasia Mutated (ATM)-deficient phenotype in pancreatic ductal adenocarcinoma, making it vulnerable to treatment that targets Checkpoint kinase 1 (CHK1). These findings support a rationale for combination therapies aimed at DNA damage response pathways.

Taken together, the studies included in this Research Topic demonstrate that epigenetic and epitranscriptomic alterations go beyond individual molecular events, instead working through intertwined networks that shape chromatin organization, RNA turnover, hypoxia signal transduction, immuno-responses, adaptation to metabolic stress, and signaling pathways involved in cellular stress. These advances support the identification of novel biomarkers and potential therapeutic targets by a deep understanding of tumor biology and molecular heterogeneity features, paving the way for more effective personalized treatments (Jamalinia and Weiskirchen, 2025).

In conclusion, we hope that this Research Topic will serve as a valuable resource for both researchers and clinicians interested in learning about the research needed to translate epigenetic discoveries into innovative diagnostic and therapeutic strategies.

## References

[B1] Dai W. Qiao X. Fang Y. Guo R. Bai P. Liu S. (2024). Epigenetics-targeted drugs: current paradigms and future challenges. Signal Transduct. Target. Ther. 9 (1), 332. 10.1038/s41392-024-02039-0 39592582 PMC11627502

[B2] Jamalinia M. Weiskirchen R. (2025). Advances in personalized medicine: translating genomic insights into targeted therapies for cancer treatment. Ann. Transl. Med. 13 (2), 18. 10.21037/atm-25-34 40438512 PMC12106117

[B3] Sadida H. Q. Abdulla A. Marzooqi S. A. Hashem S. Macha M. A. Akil A. S. A. (2024). Epigenetic modifications: key players in cancer heterogeneity and drug resistance. Transl. Oncol. 39, 101821. 10.1016/j.tranon.2023.101821 37931371 PMC10654239

[B4] Sherif Z. A. Ogunwobi O. O. Ressom H. W. (2025). Mechanisms and technologies in cancer epigenetics. Front. Oncol. 14, 1513654. 10.3389/fonc.2024.1513654 39839798 PMC11746123

